# D2/D3 dopamine supports the precision of mental state inferences and self-relevance of joint social outcomes

**DOI:** 10.1038/s44220-024-00220-6

**Published:** 2024-04-05

**Authors:** J. M. Barnby, V. Bell, Q. Deeley, M. A. Mehta, M. Moutoussis

**Affiliations:** 1 Department of Psychology, Royal Holloway, University of London, London, UK; 2 King’s College London, Cultural and Social Neuroscience Group, Department of Neuroimaging, Institute of Psychiatry, Psychology & Neuroscience, University of London, London, UK; 3 Clinical, Educational, and Health Psychology, University College London, London, UK; 4 Wellcome Centre for Human Neuroimaging, University College London, London, UK; 5 Max Planck UCL Centre for Computational Psychiatry and Ageing, University College London, London, UK

**Keywords:** Computational models, Predictive markers

## Abstract

Striatal dopamine is important in paranoid attributions, although its computational role in social inference remains elusive. We employed a simple game-theoretic paradigm and computational model of intentional attributions to investigate the effects of dopamine D2/D3 antagonism on ongoing mental state inference following social outcomes. Haloperidol, compared with the placebo, enhanced the impact of partner behaviour on beliefs about the harmful intent of partners, and increased learning from recent encounters. These alterations caused substantial changes to model covariation and negative correlations between self-interest and harmful intent attributions. Our findings suggest that haloperidol improves belief flexibility about others and simultaneously reduces the self-relevance of social observations. Our results may reflect the role of D2/D3 dopamine in supporting self-relevant mentalising. Our data and model bridge theory between general and social accounts of value representation. We demonstrate initial evidence for the sensitivity of our model and short social paradigm to drug intervention and clinical dimensions, allowing distinctions between mechanisms that operate across traits and states.

## Abstract (web summary)

In this article, the authors demonstrate that haloperidol D2/D3 dopamine antagonism contributes to flexibility in beliefs about the intentions of social partners during a sharing game.

## Main

Dysregulated striatal dopamine has been identified as a key causal component in psychosis. Influential work proposed that striatal dopamine mediates aberrant salience leading to atypical perceptual experiences^1–3^. More recent social-developmental models have highlighted the role of dopamine as a key point of convergence for a number of causal social and developmental factors, such as trauma, genetic vulnerability and cannabis use^4^. This has been supported by molecular and neuroimaging studies suggesting that developmental adversities (for example, refs. ^5,6^) increases pre-synaptic turnover of dopamine in striatal regions that may fuel the onset^7–9^ and exacerbation^10,11^ of psychosis symptoms.

Antipsychotics are the first-line treatment for psychosis and have good evidence for their efficacy^12^. Although they are thought to enact their therapeutic efficacy via D2/D3 dopamine antagonism, the exact mechanism by which their pharmacological effect reduces symptoms through the modulation of neurocognitive processes is still poorly understood. Although recent investigations into the links between striatal hyperdopaminergia and psychosis have been important in identifying important risk factors, and have offered important hypotheses for the causes of psychosis and psychotic symptoms at the neurobiological level, they have not been able to explain how they alter cognition beyond citing salience as a key mechanism. The end point of such causal pathways in psychiatry is likely to be dynamic, multidimensional, context-sensitive cognitive processes^13^. Computational modelling is an approach that allows these dynamic cognitive processes to be mathematically implemented and has the potential to more effectively connect mechanisms with psychiatric phenomenology^14,15^, offering precise accounts of complex behaviour that are more amenable to formal testing, refutation and refinement. Within this framework, dopaminergic alterations have been linked to computational processes such as belief updating^16,17^, expectations of belief volatility^18–20^ and model-based control^21^.

One particularly disabling core symptom of psychosis is paranoia, the unfounded belief that others are trying to cause you harm^22,23^. Psychologically, paranoia is characterized by heightened sensitivities to interpersonal threat^24^, attributing negative outcomes to external, personal causes^25^ and overly complex mentalising^26,27^. Developing computational theories to bridge the gap between the phenomenology and the neurocognitive mechanisms of paranoia requires particular considerations. Computational approaches in the social domain must sufficiently account for large—and often recursive—action spaces^28^. These structural principles are appropriate for psychiatric symptoms, which inherently involve alterations to interpersonal beliefs concerning the self and others^29^.

Models of intentional attributions—explicit inferences about the mental state of others—allow for analyses that are theoretically related to ongoing paranoia. Current models include mechanistic explanations for perceived changes in the harmful intent and self-interest that might motivate the actions of another. Past work suggests that high trait paranoia is associated with rigid priors about the harmful intent of partners, and a belief that a partner’s actions are not consistent with their true intentions^30,31^. Several predictions can be made concerning the influence of dopamine D2/D3 antagonism on paranoia. Synthetic in silico models^32^, neuroimaging evidence^33^, prior predictions^31^ and psychopharmacological work^21,34^ predict that D2/D3 antagonism will increase belief flexibility and improve consistency of the self’s model of others, which in turn should reduce self-relevant attributions of harmful intent following social outcomes; however, this has yet to be tested.

Although key binding sites of most antipsychotics are thought to work through their action at D2/D3 dopamine receptors, how they influence the cognitive processes of paranoia is unknown. Given the experimental evidence and synthetic predictions on the role of D2/D3 dopamine antagonism on improvements in belief updating, reductions in harmful intent, increases in prosocial behaviours, and the impact of high trait paranoia on the consistency of a self’s model of others, it follows that the mechanism of action of D2/D3 antagonism on harmful intent attributions may occur through an increase in belief flexibility and the consistency of a self’s model of others. Following from our preregistered behavioural experiment^35^, we further examine the causal influence of D2/D3 dopamine receptor antagonism on computational mechanisms governing intentional attributions within a simple game-theoretic context. Using a formal model of intentional attributions and an iterative Dictator game^30,31^, we test the impact of haloperidol, a D2/D3 antagonist and l-3,4-dihydroxyphenylalanine (l-DOPA, a presynaptic dopamine potentiator) on paranoid beliefs using past data^35^.

Primarily we assessed whether haloperidol alters key computational processes involved in mental state inferences, allowing distinctions between trait representational changes (priors) and state-learning processes (policy flexibility, uncertainty) along each attributional dimension (harmful intent and self-interest). Given the absence of any consistent descriptive effects of l-DOPA in this experiment, we modelled the data under an assumption that there would be no opposing effects on model parameters under l-DOPA versus haloperidol.

## Results

**Fig. 1:**
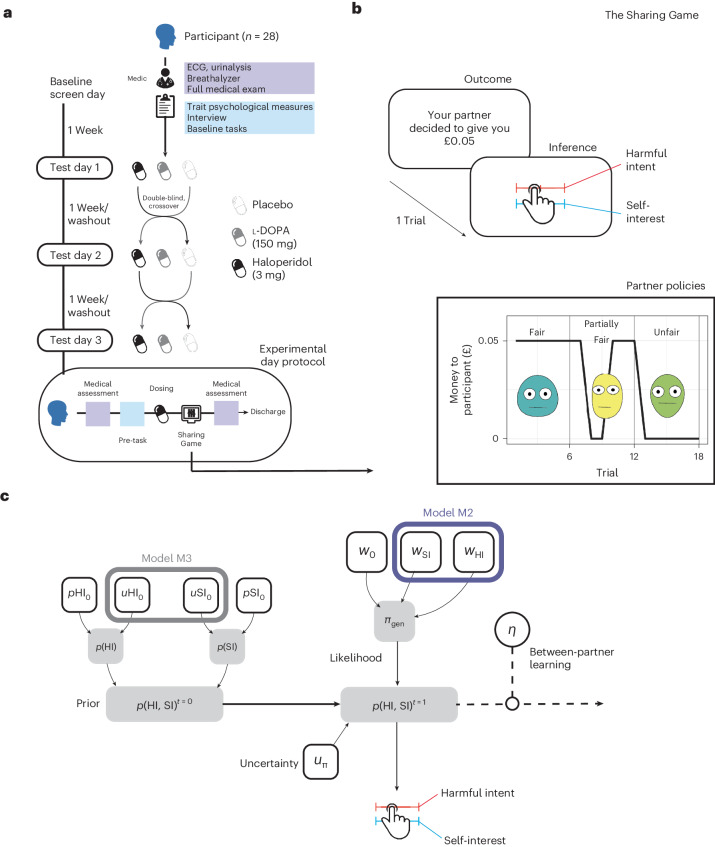
Experimental design and model space. **a**, Participants were entered into a double-blind, placebo-controlled, within-subject experimental design. ECG, electrocardiogram. **b**, Participants engaged in a three-partner version of the sharing game (inset). Here, partners were assigned the role of Dictator and, on each trial, could either take £0.10 for themselves (unfair outcome), or take £0.05 and give the participant £0.05 (fair outcome). Participant reported two types of attributional intent concerning the motivations of the partner after each outcome. These included harmful intent attributions and self-interest attributions. Partner order was randomized, and partner change was signalled. **c**, Model space used to test whether dopamine manipulations were best explained by the full model (M1), a model that constrained policy updating to a single sensitivity parameter for each attribution (M2), or a model that constrained prior uncertainty to a single parameter (M3; Table 1). Although filled objects are free parameters. Grey shaded objects are probability distributions.

Participants (*n* = 28) played a within-subjects, multi-trial modification on the Dictator game (hereafter called The Sharing Game) designed to assess paranoia^35,36^, following administration of haloperidol (3 mg), l-DOPA (150 mg) or placebo in a within-subject design (Fig. 1; see Methods for more details). After each trial of The Sharing Game, participants were asked to rate on a scale of 1–100 (initialized at 50) to what degree they believed that their partner was motivated by a desire to: (1) earn more (self-interest), and (2) reduce the participant’s bonus in the trial (harmful intent). From the participant’s perspective, the actions of the partner can be framed as either arising from motivations that concern the gain of value for the partner irrespective of the participant (other-relevant) or arising from motivations that concern the loss of value for the participant (self-relevant).

### Behavioural results

Behavioural results were published previously^35^. To summarise, when averaged over all Dictators, haloperidol caused a reduction in harmful intent attributions versus placebo (−0.17, 95% CI: −0.28, −0.05), whereas l-DOPA did not. Haloperidol also increased self-interest attributions versus placebo (0.16, 95% CI: 0.05, 0.27), whereas l-DOPA did not. Unfair and partially fair Dictators both elicited higher harmful intent (partially fair = 0.28, 95% CI: 0.16, 0.40; unfair = 0.75, 95% CI: 0.63, 0.87) and self-interest attributions (partially fair = 0.59, 95% CI: 0.63, 0.87; unfair = 1.16, 95% CI: 1.05, 1.27) versus fair Dictators.

### Model comparison and recovery

**Fig. 2:**
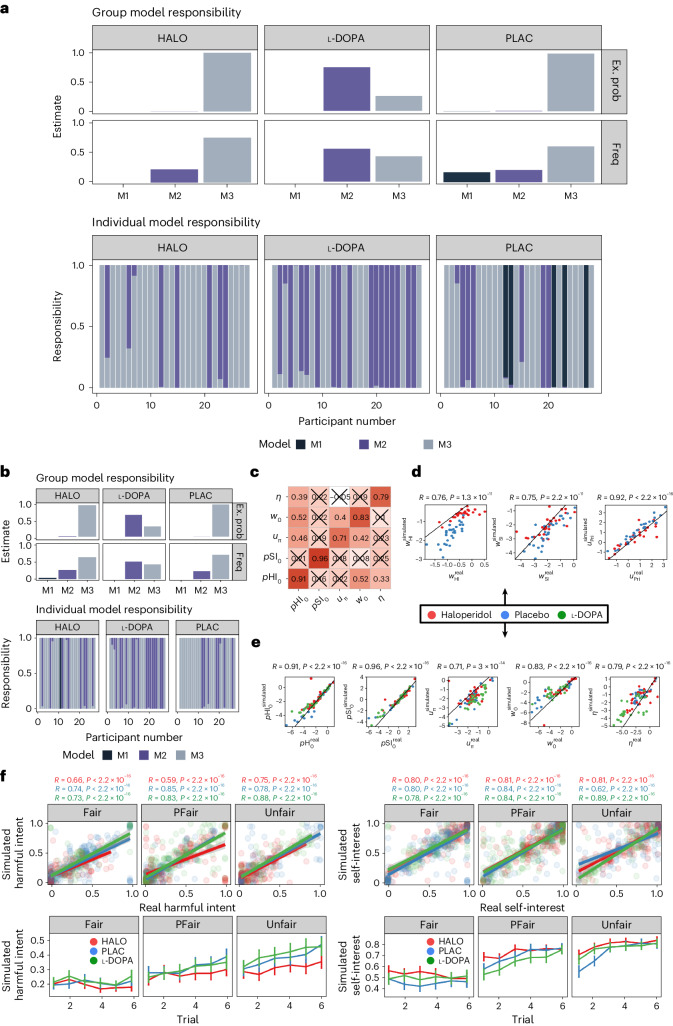
Model comparison, recovery and generative performance. **a**, Model responsibility across all three drug conditions. Greater model responsibility at the group and individual levels indicates the most likely generative model to explain the data. Ex. prob. = exceedance probability that a single model best defines group behaviour; freq = model frequency that each model is the best fitting model for participants. **b**, Model recovery. All recovery analyses used *n* = 28 synthetic participants—one for each real parameter set approximated from the data. The Hierarchical Bayesian Inference (HBI) algorithm correctly identified the correct model for most participants with trivial differences between model frequencies. **c**, Parameter recovery. Pearson correlation matrix of common parameters across all drug conditions for simulated (*y*-axis) and real (*x*-axis) data. All correlations were over 0.71 (*P*-values < 0.001). Crosses indicate non-significant associations. **d**, Parameter recovery. Individual Pearson correlations between common parameters across haloperidol and placebo conditions for simulated (*y*-axis) and real (*x*-axis) data. All correlations were over 0.71 (*P*-values < 0.001). Black lines indicate the linear model of perfect association (*r* = 1). **e**, Parameter recovery. Individual Pearson correlations between common parameters across all drug conditions for simulated (*y*-axis) and real (*x*-axis) data. Black lines indicate the linear model of perfect association (*r* = 1). **f**, Top panel: Pearson correlation (±s.e.m.) between simulated and real harmful intent (left) and self-interest (right) attributions across all Dictator policies (*n* = 28; *P*-values < 0.001). Bottom panel: simulated harmful intent (left) and self-interest (right) mean attributions (±s.e.m.) for each drug condition and Dictator policy.

Bayesian hierarchical fitting and comparison identified that, at the group level (Fig. 2a), participants under placebo and haloperidol were best fitted by model 3. This model assumed that agents use a single uncertainty over both attributional priors, but used separate likelihood weights to update their beliefs about their partners’ policy. In contrast, participants under l-DOPA were best fit by model 2. This model assumes participants hold individual uncertainties over their prior beliefs, although use the same likelihood weight to update both attributional dimensions. Importantly, model parameters under l-DOPA were not opposing haloperidol changes versus placebo, supporting behavioural analyses (Supplementary Fig. 10).

We examined model generative performance and reliability for each condition. We extracted parameters for each individual under each condition according to the model that bore the most responsibility for their behaviour (Fig. 2b). We then simulated data for each participant, with their individual-level parameters for each condition and model, and re-estimated model comparison, recovered each model, generated attributions for each trial and dictator condition, and fitted regression models for main effects. Bayesian hierarchical fitting and comparisons on simulated data demonstrated excellent similarity with group- and individual-level model responsibility and exceedance probabilities from real data (Supplementary Fig. 1a). Likewise, individual-level parameters demonstrated excellent recovery (all Pearson’s *r* values > 0.71, *P*-values ≈ 0; Supplementary Fig. 1b,c,d). Simulated and real attributions demonstrated excellent recovery across all drug and dictator conditions (all Pearson’s *r* values > 0.62, *P*-values ≈ 0; Supplementary Fig. 1e). Simulated attributions also recovered the main effects of drug and dictator conditions on attributional dynamics: haloperidol demonstrated reductions in harmful intent versus the placebo (−0.26, 95% CI: −0.36, −0.16), whereas l-DOPA did not, and haloperidol increased self-interest attributions versus the placebo (0.26, 95% CI: 0.15, 0.37), whereas l-DOPA did not.

We were most interested in examining the effect of haloperidol versus the placebo to understand the mechanism behind the observed descriptive behavioural results. As model 3 achieved group-level dominance across both placebo and haloperidol conditions, we were able to directly compare all individual-level, winning model parameters between conditions {*p*HI_0_, *p*SI_0_, *u*_Pri_, *u*_π_, *η,w*_0_, *w*_HI_, *w*_SI_} (Table 1).

**Table 1:** Winning model parameters and their role in the model

Parameter	Generative purpose
*p*HI_**0**_	Magnitude of the prior that the actions of others are generally motivated by harmful intent (HI) towards the self, *p*(HI)^*t*=0^. Increasing this parameter increases the belief that a partner is motived by harmful intent before any actions are observed.
*p*SI_**0**_	Magnitude of the prior that the actions of others are generally motivated by self-interest (SI) irrespective of the self, *p*(SI)^*t*=0^. Increasing this parameter increases the belief that a partner is motived by self-interest before any actions are observed.
*u*_Pri_	Uncertainty over priors. Increasing this parameter broadens the prior distribution of both *p*(HI)^*t*=0^ and *p*(SI)^*t*=0^.
Prior	*p*(HI)^*t*=0^ ≈ Bin(HI; *p*HI_0_, *u*_Pri_, NB) *p*(SI)^*t*=0^ ≈ Bin(SI; *p*SI_0_, *u*_Pri_, NB) *p*(HI*,*SI)^*t*=0^ = *p*(HI)^*t*=0^ *p*(SI)^*t*=0^ NB = 9
*w*_0_	Intercept of the likelihood matrix, π_gen_, which calibrates the magnitude of attributional change when a fair or unfair action is made by a partner.
*w*_HI_	Impact on beliefs that an outcome (rew) is motivated by harmful intent. Increasing this parameter leads to greater influence of a partner’s behaviour on attributions of harmful intent (belief flexibility).
*w*_SI_	Impact on beliefs that an outcome (rew) is motivated by self-interest. Increasing this parameter leads to greater influence of a partner’s behaviour on attributions of self-interest (belief flexibility).
Likelihood	π_gen_(rew *=* 0; HI*,* SI) = *σ*(*w*_0_ + [*w*_HI_ × HI*-δ*] + [*w*_SI_ × SI-*δ*]) π_gen_ (rew = 0.5; HI, SI) = 1 − π_gen_ (rew = 0;HI, SI) $\delta =\frac{\rm{NB}+1}{2}$ $\sigma (x)=\frac{1}{1+{e}^{-x}}$
Update	${\widehat{p({\rm{HI}},{\rm{SI}})}}^{t}=\frac{{\uppi }_{\rm{gen}}\left({{\mathrm{rew}};\;{\rm{HI}}},{\rm{SI}}\right){p({\rm{HI}},{\rm{SI}})}^{t-1}}{\sum _{{\rm{HI}}^{\prime} ,{\rm{SI}}^{\prime} }{\uppi }_{\rm{gen}}\left({{\mathrm{rew}};\;{\rm{HI}}}^{\prime} ,{\rm{SI}}^{\prime} \right){p({\rm{HI}}^{\prime} ,{\rm{SI}}^{\prime} )}^{t-1}}\,$
*u*_π_	The consistency with which partners were believed to act in accordance with their character. Higher values reduce consistency, causing a partner’s behaviour to have less impact on beliefs.
Consistency rule	${p\left({\rm{HI},{SI}}\right)}^{t}\propto {\widehat{p\left({\rm{HI},{SI}}\right)}}^{t\frac{1}{{\textbf{u}}{{\uppi }}}}+\xi$ $\xi =0.02/{\rm{NB}}^{2}\,$
*η*	Controls the mixture of prior and posterior beliefs used as a starting point for each new encounter. Higher values indicate more reliance on information gathered from the last encounter, rather than reverting to prior beliefs. The product from the below equation, ${\overline{p({\rm{HI},{SI})}}}^{\;t=C}$ replaces *p*(HI,SI)^*t*−1^ when beginning a new encounter.
Change point	${\overline{p({\rm{HI},{SI}})}}^{\;t=C}={p({\rm{HI},{SI}})}^{t=0} \times \left[1-{\eta }\right]+{p\left({\rm{HI},{SI}}\right)}^{t=C} \times {\eta }$ C = final action of an other in an interaction

By using model fitting procedures modellers can invert the model to approximate the parameter values that may give rise to the observed data. This includes the hidden, prior beliefs of each participant given the variance and magnitude of observed attributions. Using fitted parameter values to simulate each participant allows for generation of pseudo-experimental data—in this case, an agent’s reported intentional attributions, which we can directly compare with the real data. This also approximates the prior beliefs of each participant given the variance and magnitude of observed attributions. NB, number of bins discretizing the variable represents each attribution (in this case each distribution comprises nine bins); Bin, binomial distribution with an added precision parameter, that is, in the case of HI: *p*(HI)^*t*=0^ ≈ Bin(HI; *p*HI_0_, *u*_Pri_, NB) = *p*(HI)^*t*=0^ ≈ *B*(HI; *p*HI_0_, NB)^1/uPri^.

The bold text indicates the free parameters of interest that contribute to the equations.

### Haloperidol reduces harmful intent priors and precision

**Fig. 3:**
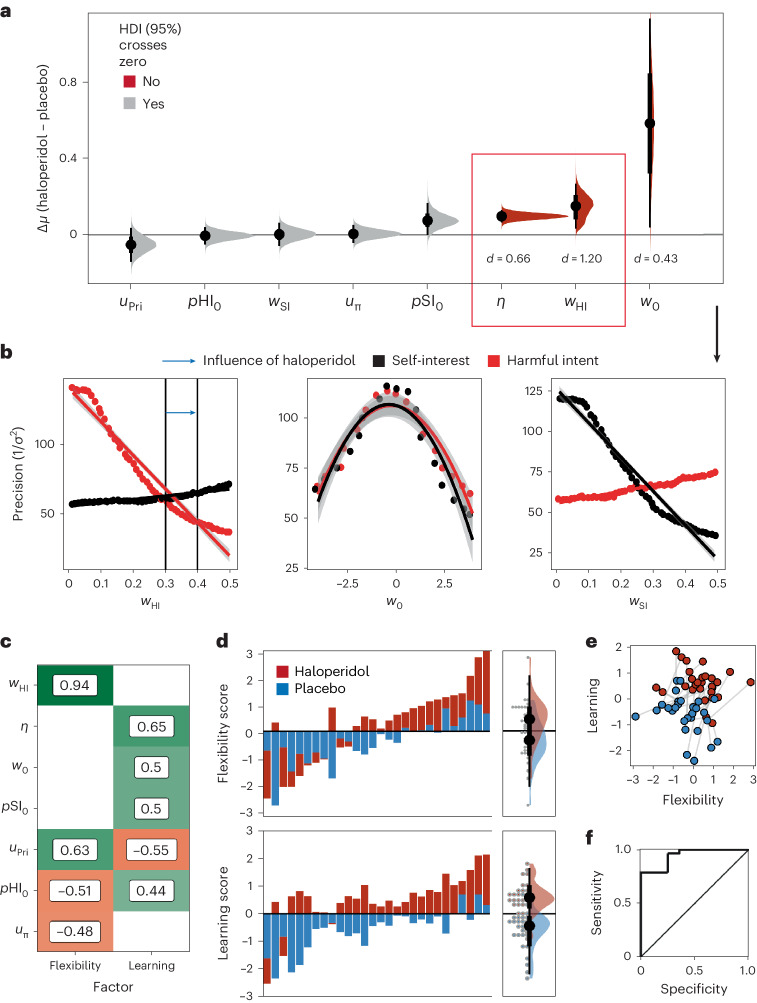
Influence of haloperidol on the winning model. **a**, Bayesian *t*-test results (*n* = 28) assessing the difference and uncertainty (median ± 95% HDI) of the change in mean parameter estimates (∆*μ*; difference in mean) between placebo and haloperidol. Red distributions indicate that the 95% high-density interval (HDI) does not cross 0, suggesting reasonable certainty that the mean difference is not an artefact of statistical noise. The *d* values indicate the median effect size (Cohen’s *d*; Supplementary Fig. 4). The red box indicates the parameters where the effect size distributions were most robust, where the 95% HDI lay outside of the region of probable equivalence with the null hypothesis. **b**, Simulations (±s.e.m.) of the marginal effect of likelihood parameters on the precision (1/*σ*^2^; inverse variance) of harmful intent (red) and self-interest (black) attributions over all trials, controlling for Dictator style. Vertical lines are indicative of the median individual parameter estimates from both haloperidol and placebo groups. The blue arrow indicates the difference from placebo to haloperidol (see Supplementary Fig. 3 for trial-wise and within-Dictator precision changes). Simulations are consistent with the notion that *w*_HI_ increases flexibility within and between contexts, accentuating smooth learning. Note that there was no significant correlation between *w*_0_, *w*_SI_ and *w*_HI_ in our parameter estimation from our real data (all *P*-values > 0.05; Supplementary Fig. 2), suggesting independent contributions from each to the attributional dynamics. **c**, Factor loading of each parameter on flexibility (factor 1) and learning (factor 2) dimensions. A loading filter of |0.4| was applied. Both of these factors can discriminate effectively between drug conditions. The *w*_SI_ term is not featured in this plot as it was not meaningfully loaded onto either factor. **d**, Factor scores (absolute value) for each individual participant (*n* = 28) for both haloperidol (red) and placebo (blue) conditions ordered from low to high. The panels on the right demonstrate the marginal loading across participants. **e**, Candyfloss plot factor scores for each individual participant. The grey lines indicate that the same participant was responsible for each connected point under placebo (blue) and haloperidol (red). **f**, Receiver operating characteristic describing the sensitivity and specificity of factors to differentiate drug conditions. Area under the curve = 0.91; sensitivity = 0.8; specificity = 0.78.

We examined the differences between individual-level parameters within subjects for haloperidol versus placebo (Fig. 3a; see Supplementary Fig. 4 for effect sizes). This suggested that haloperidol increased reliance on learning about a partner just encountered, relative to pre-existing prior beliefs about partners in general (*η*; mean diff. = 0.15, 95% HDI: 0.03, 0.26; effect size = 0.66, 95% HDI: 0.22, 1.10). Haloperidol did not influence the consistency with which partners were believed to act in accordance with their character (*u*_π_).

Haloperidol increased learning flexibility over harmful intent attributions only. Haloperidol increased the impact of partner behaviour on harmful intent attributions (*w*_HI_; mean diff. = 0.10, 95% HDI: 0.06, 0.13; effect size = 1.20, 95% HDI: 0.64, 1.75), but not over self-interest (*w*_SI_)—a partner’s actions had more impact on a participant’s beliefs about their true motivations of intentional harm. Haloperidol also caused the intercept of the policy matrix to be drawn towards 0, allowing greater updating parity for each unfair or fair partner action (*w*_0_; mean diff. = 0.58, 95% HDI: 0.01, 1.10; effect size = 0.43, 95% HDI: 0.02, 0.82). The *w*_0_ effect size should be treated with caution; the posterior distribution is within the region of practical equivalence (Supplementary Fig. 4).

We sought to further probe the model-based implications of drug differences on attributional flexibility in detail. Simulations on the marginal effect of *w*_HI_ on attributional dynamics are suggestive of its role in modulating the precision (1/*σ*^2^; inverse variance) of attributions over all trials, irrespective of Dictator policy (Fig. 3b). To establish this we used a regression model including *w*_HI_ as a linear term and *w*_0_ as a quadratic term—this was most parsimonious compared to using *w*_0_ as a linear term (Akaike Information Criterion [AIC] = 568 versus 1,123). There was a main effect of *w*_HI_ on the precision of harmful intent attributions (−6.13, 95% CI: −6.28, −5.97; effect size = −0.88, 95% CI: −0.92, −0.85). There was a small effect of *w*_0_ within the same model (−0.06, 95% CI: −0.064, −0.056, effect size = −0.11, 95% CI: −0.14, −0.08). There was a significant but small interaction of *w*_0_ and *w*_HI_ on the precision of harmful intent (−0.22, 95% CI: −0.25, −0.20; effect size = −0.05, −0.08, −0.02). Importantly, increased *w*_HI_ reduced harmful intent attributions (−0.93, 95% CI: −0.95, −0.92; effect size = −0.13, 95% CI: −0.14, −0.13) through reductions in the precision of harmful intent.

We found evidence that a greater *w*_HI_ (compare with effect of haloperidol) may reduce precision most under conditions of ambiguity. Specifically, the precision of harmful intent attributions is lower in partially fair versus fair Dictators (−0.24, −0.33, −0.15; effect size = −0.24, 95% CI: −0.33, −0.15), but unfair versus fair Dictators produced equivalent precision. Dictator policy interacts with *w*_HI_: higher *w*_HI_ is associated with lower precision under partially fair versus fair dictators (−0.77, 95% CI: −1.42, −0.42; effect size = −0.11, 95% CI: −0.21, −0.02). Thus, higher *w*_HI_ accentuates flexibility within and between partners, but most in ambiguous social contexts in which paranoia often flourishes. There was no interaction for unfair dictators versus fair dictators (Supplementary Fig. 5).

Haloperidol had no net significant influence on *p*HI_0_, *u*_Pri_ or *p*SI_0_ (Supplementary Table 1). Individual parameter analysis suggests that haloperidol has a predominant net influence on the flexibility of belief updating about a specific context (here, that of our task). Under the influence of haloperidol, participants’ assumptions of each new encounter are more amenable to change under the influence of recent encounters.

### Model covariation differentiates haloperidol from the placebo

From our analysis we can conclude that the model is accounting for the true observed data relatively well. Isolated parameter changes between conditions suggest this effect is primarily driven by increases in the impact of partner behaviour on beliefs about harmful intent, *w*_HI_ and increased learning from experience, *η*. Considered separately, these key parameters did not fully explain how the model accounted for behaviour changes induced by haloperidol (Supplementary Fig. 4). We therefore sought to identify, through exploratory factor analysis, meaningful patterns over the covariation induced by haloperidol.

We found that three factors best accounted for the data (Supplementary Fig. 9), with the first demonstrating the greatest eigenvalue (factor 1 = 2.82; factor 2 = 1.36; factor 3 = 1.13). K-fold cross-validation within a logistic model demonstrated that a two-factor solution provided the best median accuracy to discriminate between drug conditions (mean accuracy = 0.86) and had the lowest AIC (40.3; Supplementary Fig. 9). Each factor was able to predict drug condition independently (factor 1 = 1.52, 95% CI: 0.50, 2.91; factor 2 = 3.08, 95% CI: 1.72, 5.03), and there was a large effect found between conditions using Bayesian paired *t*-tests (factor 1: mean diff. = 0.76, 95% HDI = 0.37, 1.17; effect size = 0.94, 95% HDI = 0.35, 1.59; factor 2: mean diff. = 1.34, 95% HDI = 0.87, 1.85; effect size = 1.23, 95% HDI = 0.64, 1.84; Fig. 3f).

Factor 1 (flexibility; Fig. 3c) was typified by high values of *w*_HI_, and greater consistency between beliefs that a partner’s actions are indicative of their true motivations, *u*_π_. Factor 2 (learning; Fig. 3c) comprised high values of *η*, larger intercepts over the policy matrix, *w*_0_, and higher values over priors *p*SI_0_; *p*HI_0_ and *u*_Pri_ were oppositely loaded onto each factor and were likely to nullify each other in scenarios in which participants scored strongly on both (Fig. 3e). We note that *p*HI_0_ and *u*_Pri_ load with slightly more absolute value on the flexibility factor. For completeness, the third factor exclusively comprised *w*_SI_ above a cut-off of |0.4| (loading = 0.99), but it was not found to be a meaningful factor in differentiating drug scores following cross-validation and logistic model comparisons.

### Haloperidol compresses the dimensionality of partner policy

**Fig. 4:**
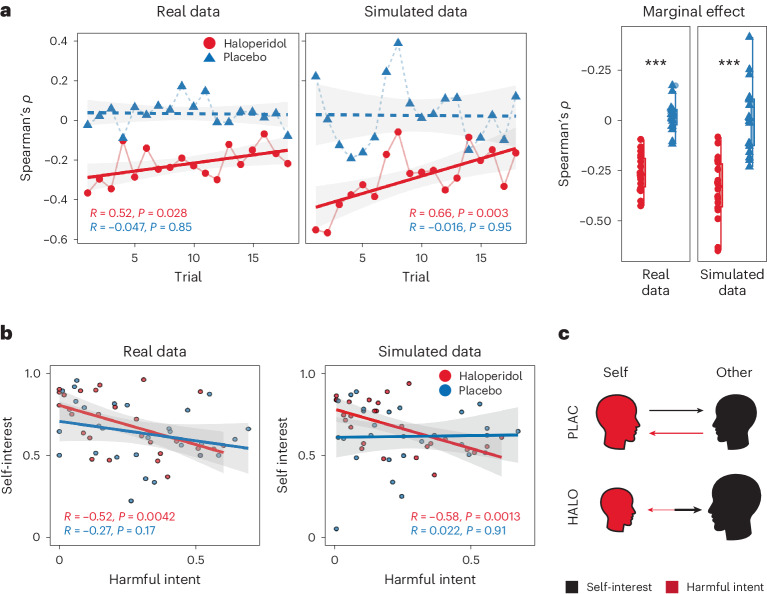
Association of mental state attributions between drug condition. **a**, In both real and simulated data (*n* = 28), haloperidol (red) versus placebo (blue) induced a trial-wise negative Pearson association (±s.e.m.) between harmful intent and self-interest, which decayed over time for both real (*R* = 0.52, *P* = 0.029) and simulated (*R* = 0.65, *P* = 0.0046) data. The right-most panel shows the marginal effect (box plots demonstrate minimum, interquartile range, median and maximum values) of trial-wise correlations between conditions. Using linear regression, we show that the difference between Pearson correlations between haloperidol and placebo was significant for both real (estimate = 2.26, SE = 0.33, *P* = 9.29 × 10^−8^) and simulated (estimate = 2.23, SE = 0.44, *P* = 1.84 × 10^−5^) data. *** = *P* < 0.001. **b**, There was a general negative Pearson association (±s.e.m.) between harmful intent and self-interest found under haloperidol for mean attributions across all 18 trials; this was not true for the placebo. **c**, Summary of main effects between drug conditions on self and other oriented intentional attributions following social outcomes. Both trial-wise and averaged associative analyses indicate that other-oriented attributions concerning self-interest of others (black), and self-oriented attributions concerning the harmful intent of others (red), are independent under the placebo (PLAC) but coupled under haloperidol (HALO). Under haloperidol this coupling is biased towards exaggeration of other-oriented attributions and diminishment of self-oriented attributions.

Finally, we explored the impact of haloperidol on attributional coupling: the dependency between intentional attributions over time. This allows analysis into the dependency of different intentional components. To calculate this we estimated Spearman correlations between harmful intent and self-interest for each trial across the sample, controlling for the type of Dictator policy affiliated. This revealed that although harmful intent and self-interest are attributed independently of one another under the placebo (mean *ρ*[s.d.] = 0.03 [0.07]; replicating ref. ^35^), under haloperidol they are negatively associated (mean *ρ*[s.d.] = −0.22 [0.08]), and this difference is significant (mean diff. = −0.26, 95% CI: −0.32, −0.20; effect size = 2.22, 95% HDI: 1.22, 3.24). This relationship was replicated using simulated model predictions (mean diff. = −0.25, 95% CI: −0.34, −0.17; effect size = −1.53, 95% HDI: −2.28, −0.78; Fig. 4a). There was evidence that the negative association induced under haloperidol decays over time (Pearson’s *r* = 0.52, *P* = 0.029). The same is not true under placebo (Fig. 4a). This interaction was not significant (regression coeff. = −0.06, 95% CI: −0.12, 0.03). In summary, haloperidol causes harmful intent and self-interest attributions to become less independent. This means that under haloperidol participants are more likely to believe someone must be more self-interested if they are perceived to be less intentionally harmful.

## Discussion

**Fig. 5:**
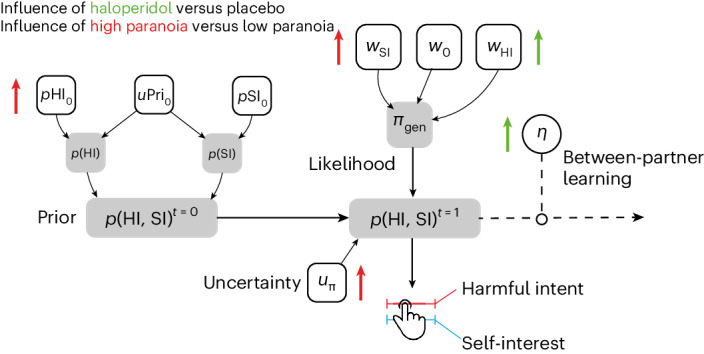
Summary of experimental parameter changes from current and past works. Experimentally observed effects on our model. The impact of haloperidol on model parameters is indicated by green arrows. Prior results from the impact of high trait paranoia^30,31^ are indicated by red arrows.

We sought to identify the computational mechanisms that explain how pharmacological alteration of dopamine function alters attributions of harmful intent—an important feature of paranoia—given our previous findings that haloperidol reduced harmful intent attributions and increased self-interest attributions in healthy participants (see ref. ^35^ for a previously published behavioural analysis). Here we tested different computational hypotheses to account more mechanistically for these effects. The data were best fit by a model utilizing a common uncertainty parameter over priors, but separate likelihood weights for updating attributions. Using this model, we found evidence that haloperidol reduced the precision of harmful intent (but not self-interest) attributions allowing more belief flexibility between partners. Haloperidol also increased the impact of learning from each encounter; participants relied less on their prior beliefs about the population as a whole. These individual parameter effects were embedded within covariational model alterations that together accounted for attributional change under haloperidol. These changes also caused self-interest and harmful-intent attributions to become negatively associated, suggesting a compression of attributions into a single interpersonal dimension under haloperidol. Together, our findings indicate haloperidol promotes flexibility regarding attributions of harmful intent to others by reducing the perceived relevance of the actions of others to the self (Fig. 5). In clinical environments this may allow space to reframe beliefs.

Our findings indicate a reduction in the influence of priors and more flexible beliefs under haloperidol. Past research links tonic dopamine at D2/D3 receptors to efficient encoding of meaningful stimuli, and Bayes optimality^33^, cognitive control^37^ and sustained attention^38^. Under the model-based, model-free control framework^39^, recent work showed that a D2/D3 antagonism increased model-based control and decision flexibility^21^, and increased belief flexibility during a trust game^34^. This may be particularly useful in ‘climbing out’ of paranoia, where one is reluctant to take in positive information about others for fear of false reassurance. At face value our results conform with previous work: under haloperidol, posteriors are more flexible and less influenced by priors, suggesting more confidence in beliefs about the motivation of partners. However, this general account does not explain why our data show asymmetric decreases in harmful intent and increases in self-interest.

One hypothesis is that haloperidol reduces the perceived self-relevance of outcomes under uncertainty. Social interaction rapidly increases the complexity of possible actions that may be taken. Humans try to reduce this uncertainty by relying on available heuristics, such as using self-preferences as an easily accessible prior belief about others^40–42^. When ambiguity increases, greater uncertainty about others^19,30,31^ and environments^20^ can increase the perception of social threat. Our analysis suggests that haloperidol may attenuate the relationship between uncertainty and attributions of harmful intent by reducing the perceived self-relevance of others’ actions; attributions of harmful intent, by definition, are inferences about the relevance of threat to the self from another. Given the role of the striatum and medial prefrontal cortex in regulating threat evaluation under stress^43^, this reduction in self-relevance may also interact with common neural implementations of self-other modelling^44^; haloperidol may modulate the degree to which information is modelled as self- or other-relevant. The degree to which D2/D3 dopamine receptor function is specific to harmful intent or all attributions that are relevant to the self (for example, altruistic intent of another) can be tested by including an extra dimension within our model; there are a number of hypotheses that can be made with such a modification (see Supplementary Fig. 7).

This pattern leads to a further, complementary proposition: haloperidol may reduce self-relevance through reductions in the complexity or depth of recursive mentalizing (how a self thinks about another’s model of the self). In general, the ability to recursively mentalize is computationally expensive^45–47^. Humans try to use cheaper strategies when possible. Recursive mentalizing is context dependent: simply, in competitive social scenarios humans are more likely to plan ahead more deeply and entertain recursive beliefs about another’s model of the self^48^. Mentalization gone awry has also been posited as a core driver of relationship difficulties in clinical populations: paranoias in borderline personality disorder and psychosis are explained as hypermentalization: the inference of overly complex mental states based on sparse data^26,27,49,50^. An alteration in mechanisms that support self-relevant mentalizing may explain our findings. This notion is consistent with reported amotivation under haloperidol (individuals are less concerned by outcomes), the role of D2/D3 receptors in promoting cognitive control^37,38^ and prior work on the causal role of D2/D3 antagonism in trust behaviours^34^; reductions in the immediate value (and therefore relevance) for the self may facilitate longer-term reciprocal trust behaviours without any need to engage deliberate reasoning about future outcomes. A core test of the hypothesis that D2/D3 dopamine is crucial for self-relevant, recursive mentalization is to use models of hierarchical mentalization in future experiments that allow estimation of recursive depth in joint social contexts.

The data presented here may be relevant beyond psychiatry. In behavioural economics, there have been several studies on the role of dopamine, reward and decision making in both social and non-social contexts^51^. Increasing dopamine availability has been shown to increase risky non-social decisions when self-gain is at stake^52^, suggesting that dopamine may inflate the attributed value of outcomes to the self. Our data imply that this role of dopamine in modulating monetary value to the self may reflect a broader role in representing the self-relevance of stimuli. The direction of this relationship (self-relevance precedes self-value, or vice versa) is a fruitful target for future research. Our data may also be relevant to the role of dopamine in moral behaviour. In one study, boosting D2/D3 dopamine with pramipexole reduced generosity, especially with close others^53^. Our data complement this work, suggesting that D2/D3 dopamine is involved in calibrating the valuation of self-gain in social decision-making.

On a theoretical level, our formal model distinguishes between computational changes that result from prior representational biases (for example, higher trait paranoia) and acute state changes during social interaction where potential harm from others is a possibility (Fig. 5). Previous modelling with the same task^30^—or a reversal variant of the task^31^—provided evidence that trait paranoia increases the magnitude of priors over harmful intent, the subsequent increase in the belief that the actions of others are not reflective of their true motivations and a reduced willingness to believe that changes to a partner’s behaviour are motivated by changes to their harmful intent. Naturally, this suggested that prior representations bias how social behaviour is interpreted. On the other hand, the present models suggest that haloperidol acts through increased reliance and impact of likelihoods on the formation of beliefs. Creating phenomenologically plausible formal models that are sensitive to different explanations of behavioural data has been a core aspiration of computational psychiatry^13,14^. Models such as ours may be useful in distinguishing between longer-term development and near-term alterations in learning that may explain paranoia. Model parameters are constant on the timescale of tasks while potentially evolving at the timescale of personal development, illness and recovery, whereas learning and inference can be dissected on the timescale of task conditions and trials. Much like prior work distinguishing interventions of representational change (psychotherapy) and emotion modulation (antidepressants^54^), our model may support similar distinctions following intervention. We thus hypothesize that successful therapeutic use of haloperidol in paranoia will be associated with large changes in likelihood parameters described above but may leave intact, at least in the short term, prior beliefs about the harmful intent of others; D2/D3-independent processes may underpin ongoing vulnerability and may require further psychosocial learning. Our task may only pick up long-term representational (prior) changes following extended pharmacological therapy, or in combination with psychological therapy.

We note some limitations. First, we did not use a patient population, which means the extent to which the findings generalize to a population with persecutory delusions—rather than non-delusional paranoia—remains unclear. Likewise, in this first study we only included males to avoid hormonal heterogeneity, which might affect drug response and indeed the precise expression of dopaminergic mechanisms^55^. However, this important limitation must be addressed in future studies with studies powered to examine the computational structure of antipsychotic medication in people of different hormonal status and gender. Second, we did not include any non-social comparator (for example, model-based decision making or volatile environments) when assessing the role of haloperidol on cognition. This leaves a divide between how dopamine influences non-social cognition and mental state inferences. Past work suggests some shared variance between more foundational computations (for example, decision temperature, belief updating) and paranoia^20,31,33^. Replicating the present work with non-social comparators of our social task (for example, using a slot machine partner) may help understand the relations between formal theories of general decision making, and how this is expressed at a recursive and intentional level in the same individuals. Third, we did not use a design that probes how dopamine may facilitate generalization of social knowledge outside of our game theory task. Prior work has demonstrated that representations about learned partners can pass on from one context to another^45^; once a representation is learned using computationally intensive resources, a cheaper, heuristic model can be used. This relates to the question of whether an associative model of updating may be more efficient once a policy is known, and given our findings, whether haloperidol causes a faster transition. Finally, despite the difference in model responsibility, we did not find any influence of l-DOPA on behaviour. This may be due to an insufficient dose or translation of l-DOPA leading to an increase in dopamine release, or the unspecific postsynaptic binding that may result from any successfully increased dopamine release as a consequence of l-DOPA.

## Methods

### Participants

This study was approved by KCL ethics board (HR-16/17-0603). All data were collected between August 2018 and August 2019. Participants were recruited through adverts in the local area, adverts on social media, in addition to adverts circulated via internal emails. Participants provided written informed consent to take part.

Eighty-six participants were preliminarily phone screened; 35 participants were given a full medical screen; 30 healthy males were recruited to take part in the full procedure; 2 failed to complete all experimental days, leaving 28 participants for analysis (age (mean[s.d.]) = 29.21[8.61]). Inclusion criteria were that participants were healthy males, between the ages of 18 and 55. Participants were excluded if they had any evidence or history of clinically significant medical or psychiatric illness; if their use of prescription or non-prescription drugs was deemed unsuitable by the medical team; if they had any condition that may have inhibited drug absorption (for example gastrectomy); a history of harmful alcohol or drug use determined by clinical interview; use of tobacco or nicotine-containing products in excess of the equivalent of five cigarettes per day; a positive urine drug screen; or were unwilling or unable to comply with the lifestyle guidelines. Participants were excluded who, in the opinion of the medical team and investigator, had any medical or psychological condition, or social circumstance, that would impair their ability to participate reliably in the study, or who may increase the risk to themselves or others by participating. Some of these criteria were determined through telephone check for non-sensitive information (age, gender, general understanding of the study and overall health) before their full screening visit. Participants were paid £100 for successful completion of all experimental days, and £20 if they failed screening and were subsequently excluded.

### Procedure

This study was part of a larger study that assessed the role of dopaminergic modulation on personality, beliefs and social interaction. Here we focus on the role of dopamine antagonism and pre-synaptic increases in the attribution of mental state inferences during a Dictator game (described below; see Fig. 1a).

The full procedure for participant screening is documented in a prior publication^35^. Briefly, participants who passed the brief phone screening were invited to attend an on-site screening day (see above). Participants were tested for drugs of abuse (SureScreen Diagnostics) and alcohol (breath test) prior to each experimental day and were excluded if any test was positive. Participants were given at least 7 days, but no more than 2 months, in between experimental days to allow for drug washout.

On experimental days, participants were randomized to be initially administered either a placebo or 3 mg haloperidol in two capsules, and 10 mg of domperidone (to reduce known side effects of vomiting and nausea that can appear in some recipients) in one capsule (3 caps total). After 30 min, participants were dosed a second time with either 150 mg of co-beneldopa (herein referred to as l-DOPA) or placebo in two capsules. Participants would never receive haloperidol and l-DOPA in the same day.

### The Sharing Game

Participants were asked to play a within-subjects, multi-trial modification on the Dictator game design used in previous studies to assess paranoia^35,36^, hereafter called ‘The Sharing Game’ (Fig. 1). In the game, participants played six trials against three different types of partner who are assigned the role of Dictator. In each trial, participants were told that they have a total of £0.10 and their partner (the Dictator) had the choice to take half (£0.05) or all (£0.10) the money from the participant. Partner policies were one of three types: always take half of the money, have a 50:50 chance to take half or all of the money, or always take all of the money. These policies were labelled as fair, partially fair and unfair, respectively. The order that participants were matched with partners was randomized. Each partner had a corresponding cartoon avatar with a neutral expression to support the notion that each of the six trials was with the same partner.

After each trial, participants were asked to rate on a scale of 1–100 (initialized at 50) to what degree they believed that their partner was motivated (1) by a desire to earn more (self-interest), and (2) by a desire to reduce their bonus in the trial (harmful intent). From the participants perspective, the actions of the partner can be framed as either arising from motivations that concern the gain of value for the partner irrespective of the participant (other-relevant) or arising from motivations that concern the loss of value for the participant (self-relevant).

After making all 36 attributions (two trial attributions for each of the six trials over three partners), participants were put in the role of the Dictator for six trials—whether to make a fair or unfair split of £0.10. Participants were first asked to choose an avatar from nine different cartoon faces before deciding on their six different splits. These Dictator decisions were not used for analysis but were collected to match subsequent participants with decisions from real partners. Participants were paid a baseline payment for their completion, plus any bonus they won from the game.

### Analysis

Behavioural data have been previously published^35^. Here, we apply three computational hypotheses which could explain the data, centred around a Bayesian model^31^ developed to explain mental state inference dynamics during social observation, where recursive, strategic social action is not a process of interest^29^. We note that previous work showed a Bayesian instantiation of this attributional model outperformed associative model variants^31^. Model 1 allowed separate uncertainties and likelihood weights for each attribution, identical to our prior work^31^; this model demonstrated that trait paranoia increased belief rigidity and self-other inconsistency, and by extension, may serve as a useful assay to test the mechanisms of haloperidol which is theorized to reduced paranoia. In line with general theories of belief updating^56^, Model 2 hypothesized that beliefs would be updating with the same likelihood weight. Model 3 hypothesized that prior beliefs share a single uncertainty free parameter over each distribution, allowing for a simpler hypothesis that prior uncertainties may be represented by a single dimension, giving a more parsimonious account of the data. Descriptions of the parameters within the winning model are in Table 1.

The winning model uses eight parameters that calibrate an agent’s initial and ongoing beliefs about others. It encodes the agent’s prior expectations of harm, *p*HI_0_, and self-interest, *p*SI_0_, and the certainty of these expectations, *u*_Pri_. Three parameters implement the agent’s internal likelihood of a partner acting with self-interest or harm based on their behaviour, influencing belief updates (*w*_0_, *w*_HI_, *w*_SI_). A noise parameter (*u*_π_) indicates the agent’s uncertainty over the representation of their partner. The model also includes a belief persistence parameter, *η*, for agents to either persist with their most recent beliefs or re-set them to the prior expectations (above) upon encountering new partners, with higher values indicating less resetting. See Table 1 for further details.

All computational models were fitted using an HBI algorithm which allows hierarchical parameter estimation while assuming random effects for group and individual model responsibility^57^. This process is shown to be most robust to outliers versus non-hierarchical inference or standard hierarchical inference with fixed effects, and minimizes parameter and model confusion^57^. Parameters were estimated using the HBI in native space drawing from broad priors (*μ*_*m*_ = 0, *σ*_*m*_ = 6.5; where *m* = {*m*_1_, *m*_2_, *m*_3_}). This process was run independently for each drug condition due to the dependency of observations between conditions (the same participants were in each condition). Parameters were transformed into model-relevant space for analysis. All models and hierarchical fitting was implemented in Matlab (Version R2022B). All other analyses were conducted in R (v.4.2.3; x86_64 build) running on Mac OS (Ventura v.13.0). All statistics are reported as: (*X*, 95% CI: *Y*, *Z*), where *X* is the regression coefficient, and *Y* and *Z* are the 95% lower and upper CIs, respectively. All dependent regressors were centred and scaled. To consider the uncertainty of estimates we conducted Bayesian paired sample *t*-tests to assess individual-level parameter changes. This used JAGS as a backend MCMC sampler^58^; differences in the mean are additionally reported with their corresponding effect sizes (Cohen’s *d*) and posterior 95% HDI. The raw output of this is listed in Supplementary Table 1. Bayesian paired sample *t*-tests were also used to assess differences between attributional coupling over time. To note, in the original behavioural analysis^35^ we excluded one extra participant due to their extreme trait psychometric paranoia score (leaving 27 participants); however trait paranoia was not the subject of this analysis, and hierarchical model fitting constrains group behaviour during parameter estimation. Nevertheless, for transparency, we include analytic estimates with the original 27 individual included for comparison. This did not change conclusions (Supplementary Table 2).

We also sought to examine model covariance. Exploratory factor analysis used oblique rotation, including all parameter estimates for each individual within placebo and haloperidol conditions. Optimal factors were determined from observation of the scree plot and cross-validated model accuracy (Supplementary Fig. 9). Cross-validation used ten folds with three repeats within a logistic general linear model. Parameter loadings and individual factor scores >|0.4| were retained for analysis.

### Reporting summary

Further information on research design is available in the Nature Portfolio Reporting Summary linked to this article.

### Supplementary information

Supplementary Information Supplementary Figs. 1–10, and Tables 1 and 2.

Reporting Summary

## Data Availability

All data are freely available online: https://github.com/josephmbarnby/Barnby_etal_2023_D2D3Modelling (ref. ^59^).

## Code availability

All analysis codes are freely available online: https://github.com/josephmbarnby/Barnby_etal_2023_D2D3Modelling (ref. ^59^).

## References

[1] Howes OD, Kapur S (2009). The dopamine hypothesis of schizophrenia: version III—the final common pathway. Schizophr. Bull..

[2] Kapur S (2004). How antipsychotics become anti-‘psychotic’—from dopamine to salience to psychosis. Trends Pharmacol. Sci..

[3] Kapur S, Mizrahi R, Li M (2005). From dopamine to salience to psychosis—linking biology, pharmacology and phenomenology of psychosis. Schizophr. Res..

[4] Howes OD, Murray RM (2014). Schizophrenia: an integrated sociodevelopmental-cognitive model. Lancet.

[5] Dahoun T (2019). The relationship between childhood trauma, dopamine release and dexamphetamine-induced positive psychotic symptoms: a [^11^C]-(+)-PHNO PET study. Transl. Psychiatry.

[6] Egerton A (2016). Adversity in childhood linked to elevated striatal dopamine function in adulthood. Schizophr. Res..

[7] Howes OD (2011). Dopamine synthesis capacity before onset of psychosis: a prospective [^18^F]-DOPA PET imaging study. Am. J. Psychiatry.

[8] Howes O (2011). Progressive increase in striatal dopamine synthesis capacity as patients develop psychosis: a PET study. Mol. Psychiatry.

[9] Jauhar S (2019). Determinants of treatment response in first-episode psychosis: an ^18^F-DOPA PET study. Mol. Psychiatry.

[10] Laruelle M (1996). Single photon emission computerized tomography imaging of amphetamine-induced dopamine release in drug-free schizophrenic subjects. Proc. Natl Acad. Sci. USA.

[11] Laruelle M, Abi-Dargham A (1999). Dopamine as the wind of the psychotic fire: new evidence from brain imaging studies. J. Psychopharmacol..

[12] Schneider-Thoma J (2022). Comparative efficacy and tolerability of 32 oral and long-acting injectable antipsychotics for the maintenance treatment of adults with schizophrenia: a systematic review and network meta-analysis. Lancet.

[13] Hitchcock PF, Fried EI, Frank MJ (2022). Computational psychiatry needs time and context. Ann. Rev. Psychol..

[14] Huys QJ, Maia TV, Frank MJ (2016). Computational psychiatry as a bridge from neuroscience to clinical applications. Nat. Neurosci..

[15] Montague PR, Dolan RJ, Friston KJ, Dayan P (2012). Computational psychiatry. Trends Cogn. Sci..

[16] Adams RA, Stephan KE, Brown HR, Frith CD, Friston KJ (2013). The computational anatomy of psychosis. Front. Psychiatry.

[17] Ashinoff BK, Singletary NM, Baker SC, Horga G (2022). Rethinking delusions: a selective review of delusion research through a computational lens. Schizophr. Res..

[18] Diaconescu AO, Wellstein KV, Kasper L, Mathys C, Stephan KE (2020). Hierarchical Bayesian models of social inference for probing persecutory delusional ideation. J. Abnormal Psychol..

[19] Hauke DJ (2023). Aberrant perception of environmental volatility during social learning in emerging psychosis. Comput. Psychiatry.

[20] Reed EJ (2020). Paranoia as a deficit in non-social belief updating. eLife.

[21] Mikus N (2022). Effects of dopamine D2/3 and opioid receptor antagonism on the trade-off between model-based and model-free behaviour in healthy volunteers. eLife.

[22] Freeman D (2016). Persecutory delusions: a cognitive perspective on understanding and treatment. Lancet Psychiatry.

[23] Brakoulias V, Starcevic V (2008). A cross-sectional survey of the frequency and characteristics of delusions in acute psychiatric wards. Australasian Psychiatry.

[24] Raihani NJ, Bell V (2019). An evolutionary perspective on paranoia. Nat. Hum. Behav..

[25] Bentall RP, Kinderman P, Kaney S (1994). The self, attributional processes and abnormal beliefs: towards a model of persecutory delusions. Behav. Res. Therapy.

[26] Fonagy P, Target M (1996). Playing with reality: I. Theory of mind and the normal development of psychic reality. Int. J. Psychoanal..

[27] Alon, N., Schulz, L., Dayan, P., & Barnby, J. M. Between prudence and paranoia: theory of mind gone right, and wrong. In *First Workshop on Theory of Mind in Communicating Agents* (PMLR, 2023).

[28] FeldmanHall O, Nassar MR (2021). The computational challenge of social learning. Trends Cogn. Sci..

[29] Barnby JM, Dayan P, Bell V (2023). Formalising social representation to explain psychiatric symptoms. Trends Cogn. Sci..

[30] Barnby JM, Bell V, Mehta MA, Moutoussis M (2020). Reduction in social learning and increased policy uncertainty about harmful intent is associated with pre-existing paranoid beliefs: evidence from modelling a modified serial dictator game. PLoS Comput. Biol..

[31] Barnby JM, Mehta MA, Moutoussis M (2022). The computational relationship between reinforcement learning, social inference, and paranoia. PLoS Comput. Biol..

[32] Adams RA, Vincent P, Benrimoh D, Friston KJ, Parr T (2022). Everything is connected: inference and attractors in delusions. Schizophr. Res..

[33] Nour MM (2018). Dopaminergic basis for signaling belief updates, but not surprise, and the link to paranoia. Proc. Natl Acad. Sci. USA.

[34] Mikus, N. et al. Blocking D2/D3 dopamine receptors increases volatility of beliefs when we learn to trust others. *Nat. Commun.* **14**, 4049 (2022). 10.1038/s41467-023-39823-5 PMC10329681 37422466

[35] Barnby, J. M., Bell, V., Deeley, Q. & Mehta, M. A. Dopamine manipulations modulate paranoid social inferences in healthy people. *Transl. Psychiatry* **10**, 214 (2020). 10.1038/s41398-020-00912-4 PMC7335741 32624569

[36] Barnby JM (2020). Paranoia, sensitization and social inference: findings from two large-scale, multi-round behavioural experiments. R. Soc. Open Sci..

[37] Cools R, D’Esposito M (2011). Inverted-U-shaped dopamine actions on human working memory and cognitive control. Biol. Psychiatry.

[38] Saeedi H, Remington G, Christensen BK (2006). Impact of haloperidol, a dopamine D2 antagonist, on cognition and mood. Schizophr. Res..

[39] Daw ND, Niv Y, Dayan P (2005). Uncertainty-based competition between prefrontal and dorsolateral striatal systems for behavioral control. Nat. Neurosci..

[40] Andersen, S. M. & Chen, S. The relational self: an interpersonal social-cognitive theory. *Psychol. Rev.* **109**, 619–645 (2002). 10.1037/0033-295x.109.4.619 12374322

[41] Barnby, J. M., Raihani, N. & Dayan, P. Knowing me, knowing you: interpersonal similarity improves predictive accuracy and reduces attributions of harmful intent. *Cognition* **225**, 105098 (2022). 10.1016/j.cognition.2022.105098 35349872

[42] Tarantola T, Kumaran D, Dayan P, De Martino B (2017). Prior preferences beneficially influence social and non-social learning. Nat. Commun..

[43] Vaessen T, Hernaus D, Myin-Germeys I, van Amelsvoort T (2015). The dopaminergic response to acute stress in health and psychopathology: a systematic review. Neurosci. Biobehav. Rev..

[44] Nicolle A (2012). An agent independent axis for executed and modeled choice in medial prefrontal cortex. Neuron.

[45] Devaine, M., Hollard, G. & Daunizeau, J. Theory of mind: did evolution fool us? *PLoS ONE* **9**, e87619 (2014). 10.1371/journal.pone.0087619 PMC3914827 24505296

[46] Guennouni I, Speekenbrink M (2022). Transfer of learned opponent models in zero sum games. Comput. Brain Behav..

[47] de Weerd H, Diepgrond D, Verbrugge R (2018). Estimating the use of higher-order theory of mind using computational agents. B. E. J. Theor. Econ..

[48] Goodie AS, Doshi P, Young DL (2012). Levels of theory‐of‐mind reasoning in competitive games. J. Behav. Decis. Mak..

[49] Sharp, C. in *Handbook of Borderline Personality Disorder in Children and Adolescents* 211–225 (Springer, 2014).

[50] Fonagy P, Bateman AW (2006). Mechanisms of change in mentalization-based treatment of BPD. J. Clin. Psychol..

[51] Cox J, Witten IB (2019). Striatal circuits for reward learning and decision-making. Nat. Rev. Neurosci..

[52] Rutledge RB, Skandali N, Dayan P, Dolan RJ (2015). Dopaminergic modulation of decision making and subjective well-being. J. Neurosci..

[53] Oroz Artigas, S. et al. Enhancement in dopamine reduces generous behaviour in women. *PLoS ONE* **14**, e0226893 (2019). 10.1371/journal.pone.0226893 PMC6938376 31891605

[54] Nord CL (2021). Neural effects of antidepressant medication and psychological treatments: a quantitative synthesis across three meta-analyses. Brit. J. Psychiatry.

[55] Seeman MV (2021). The pharmacodynamics of antipsychotic drugs in women and men. Front. Psychiatry.

[56] Erdmann T, Mathys C (2022). A generative framework for the study of delusions. Schizophr. Res..

[57] Piray P, Dezfouli A, Heskes T, Frank MJ, Daw ND (2019). Hierarchical Bayesian inference for concurrent model fitting and comparison for group studies. PLoS Comput. Biol..

[58] Bååth, R. Bayesian first aid: a package that implements Bayesian alternatives to the classical *.test functions in R. In *Proc.**Use R! 2014*—*The International R User Conference* (UCLA, 2014).

[59] josephmbarnby/Barnby_etal_2023_D2D3Modelling. *GitHub* https://github.com/josephmbarnby/Barnby_etal_2023_D2D3Modelling (2024).

